# Rapid and Environmentally‐Friendly Synthesis of Thiazolidinone Analogues in Deep Eutectic Solvent Complemented with Computational Studies

**DOI:** 10.1002/open.202400198

**Published:** 2024-10-25

**Authors:** Gobind Kumar, Pule Seboletswe, Nontobeko Gcabashe, Sanjeev Dhawan, Neha Manhas, Gaurav Bhargava, Rupesh Kumar, Parvesh Singh

**Affiliations:** ^1^ School of Chemistry and Physics University of KwaZulu Natal P/Bag X54001 Westville, Durban 4000 South Africa; ^2^ Department of chemical sciences I. K. Gujral Punjab Technical University Kapurthala Punjab 144603 India

**Keywords:** Deep eutectic solvent, Rhodanine, Green chemistry, DFT, Heterocyclic compounds, Benzylidene-2-thioxothiazolidinone

## Abstract

A greener, safer, and more efficient methodology for the synthesis of (Z)‐5‐benzylidene‐2‐thioxothiazolidin‐4‐ones (**3 a**–**u**) and (*Z*)‐5‐benzylidenethiazolidine‐2,4‐diones (**4 a**–**i**) has been developed. The deep eutectic solvent (DES) ZnCl_2_/urea used as a greener solvent as well as a catalyst in this study accelerated the condensation of rhodanine and thiazolidine‐2,4‐dione with different aldehydes to afford the target scaffolds in excellent yields (88‐98 %). The reaction methodology adopted offered significant advantages such as mild reaction conditions, functional group tolerance, quick reaction time, column‐free isolation, catalytic recyclability, and applicability to gram‐scale production. Moreover, density function theory calculations were carried out to investigate the global reactivity and stability profiles of these compounds. Finally, the green metrics analysis supported the greener nature of the present methodology.

## Graphical abstract

Deep eutectic solvent (ZnCl_2_/urea) facilitates the condensation of rhodanine or thiazolidine‐2,4‐dione with various aldehydes. The present protocol offers remarkable advantages including quick reaction, high to excellent yield, column free, recyclable solvent and catalyst. The green metrics analysis suggests the present methodology to be eco‐friendly.

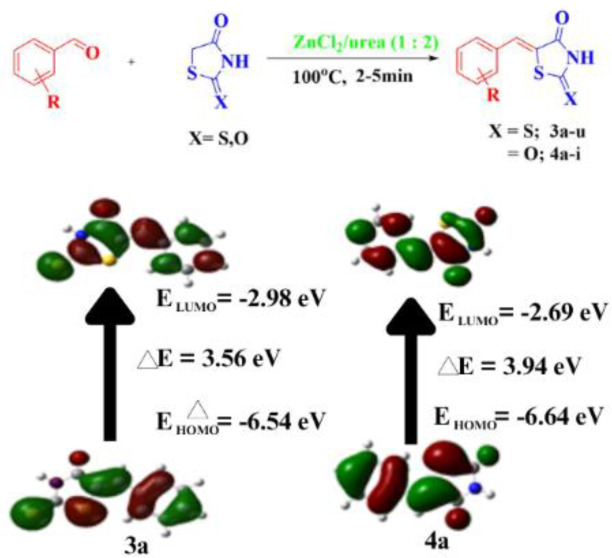

## Introduction

Developing efficient, green, and environmentally friendly methodologies for synthesizing widely applicable chemical scaffolds by using readily available compounds is a significant challenge for researchers in the field of synthetic chemistry.[^1^, ^2^, ^3^, ^4^] Several toxic catalysts and solvents being used in the synthetic chemistry, chemical, and pharmaceutical industries are potential environmental and health hazards.[^5^, ^6^, ^7^] Hence, researchers and chemists are regularly prompted to make concerted efforts to search for more efficient and environmentally friendly solvents and catalysts to reduce the environmental and health impact of such toxic materials and chemical processes.[^8^, ^9^]

Various green and eco‐friendly solvents such as glycerol,^[10]^ supercritical fluids,^[11]^ bio‐based solvents,^[12]^ water,^[13]^ and ionic liquids (ILs) have been explored in past decades.^[14]^ However, these solvents have also been associated with several limitations and shortcomings, which have hindered their widespread implementation in synthetic chemistry, thus necessitating innovative, versatile, and eco‐friendly solvents specialized for supercritical fluid applications.^[15]^ Ionic liquids, offering remarkable properties such as chemical and thermal stability, recyclability, low volatility, and non‐flammability, have gained significant attention as potential green solvents.[^16^, ^17^, ^18^, ^19^] However, many challenges are associated with their production, including the complexities of purification, moisture and air sensitivity, and the complex use of organic solvents during reactions.^[20]^ Furthermore, their high toxicity and low biodegradability have restricted their application and compromised atom economy and environmental sustainability.[^21^, ^22^]

Contrary to traditional ILs, deep eutectic solvents (DESs) exhibit several advantages, offering remarkable similarities with traditional ILs at the same time.[^23^, ^24^] The DESs have similar physical properties as traditional ILs, but they have different chemical properties and offer a wide range of applications with respect to ILs.[^25^, ^26^, ^27^] The DESs can be easily prepared by mixing precise molar compositions of two or more components at moderate temperatures.^[28]^ DESs have shown dominance over ILs as they offer significant properties such as low‐toxicity, cost‐effectiveness, ease of accessibility, suitability for use without rigorous purification, biodegradability, straightforward work‐up procedures, and the capacity to function autonomously without additional organic solvents.[^29^, ^30^, ^31^, ^32^] DESs have demonstrated a wide range of applications in different fields including (i) metallurgy and electrodeposition, (ii) separations and gas capture, (iii) power system and battery technology, (iv) biomass processing, biomolecular structure, folding, and stability, (v) genomics/fundamentals of nucleic acids, (vi) pharmaceuticals and medical research and (vii) biocatalyst and solvent in organic reactions.[^33^, ^34^, ^35^, ^36^] Moreover, DESs have also been used to develop new drug delivery systems (e. g. therapeutic deep eutectic solvent) to enhance the solubility issues of active pharmaceutical ingredients.[^37^, ^38^, ^39^]

**Figure 1:**
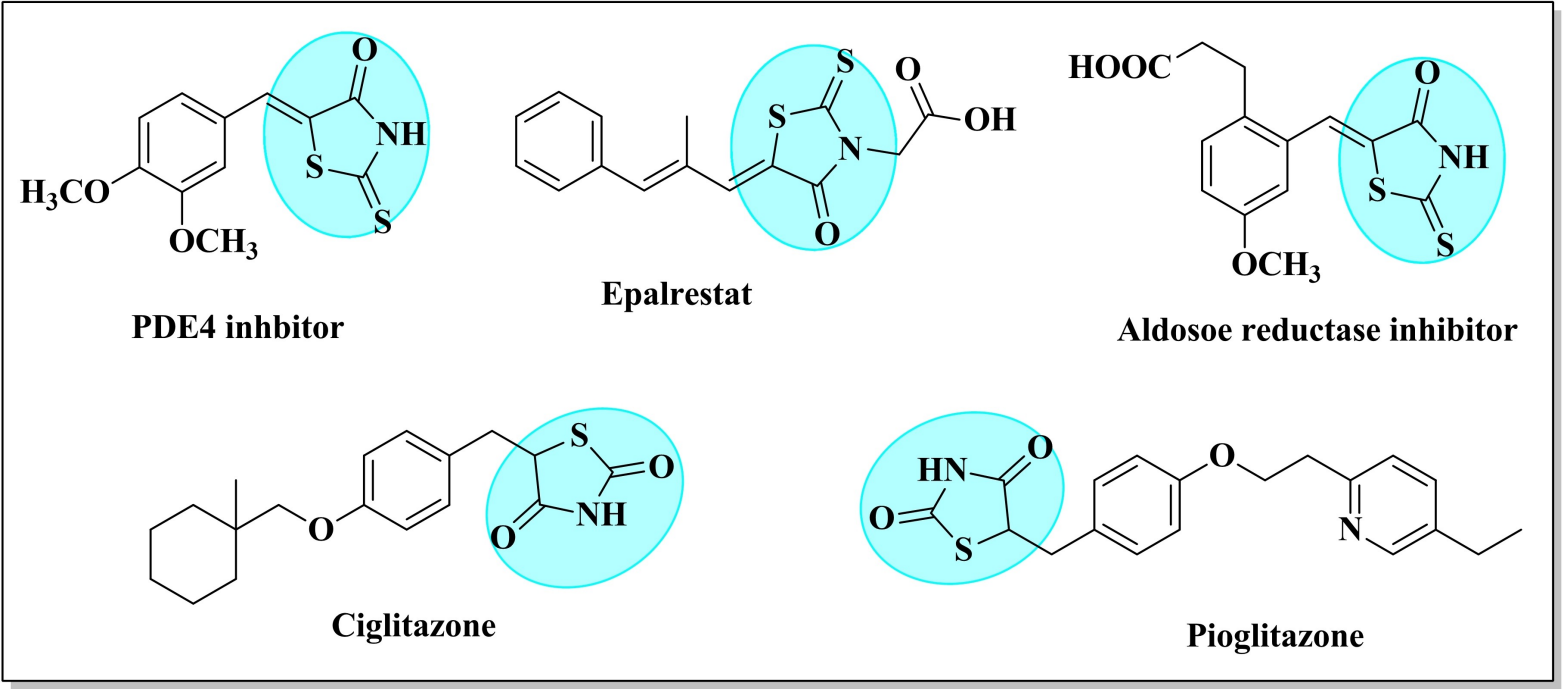
Some important biologically active rhodanine and thiazolidine‐2,4‐dione analogues.

Thiazolidines, particularly rhodanine and thiazolidine‐2,4‐dione, have gained significant attention owing to their diverse pharmacological activities (Figure 1), including antidiabetic, antimicrobial, antimalarial, antiviral, anticonvulsant, anti‐inflammatory, antitubercular properties, and their role as thyroid hormone receptor antagonists.[^40^, ^41^, ^42^, ^43^] Additionally, these derivatives serve as small‐molecule inhibitors of various key cellular enzymes such as 15‐hydroxyprostaglandin dehydrogenase (15‐PGDH),^[44]^ HIV‐1,^[45]^ aldose reductase,^[46]^ and tyrokinase.^[47]^ They also exhibit inhibitory effects on diverse targets, such as HCV protease, β‐lactamase, JNK‐stimulating phosphatase‐1 (JSP‐1), aldose reductase, tyrosine phosphatases, and PMT1 mannosyl transferase.^[44]^

Accordingly, efforts have been made to find simple and efficient methods for the synthesis of different thiazolidinone analogues. The literature studies particularly revealed the employment of condensation reaction between different aldehydes and rhodanine and its analogues in this direction. The different catalytic systems and reaction conditions, such as piperidine, TS‐1, [bmim][OH], Bu_4_NOH, tetrabutylammonium bromide (TBAB), sodium acetate, acetic and PEG‐300, Iodine, piperidinium benzoate, have been reported for the production of these compounds (Table 4).[^48^, ^49^, ^50^, ^51^, ^52^] However, the existing methods suffer from several drawbacks, such as prolonged reaction times, environmentally unfavorable solvents, non‐recyclability of catalysts, and frequent low yields of the target scaffolds. Therefore, there is still room to improve the reaction conditions regarding quick reaction time, easy work‐up, recyclability of catalyst and eco‐friendly solvent.

To overcome these challenges, the development of new methodologies in current organic synthesis, aligned with Green Chemistry principles, is crucial for sustainable chemistry. Following the same principles, our research group has explored the potential of DESs, such as green solvents and catalysts, in synthesizing many heterocyclic scaffolds in the past few years. In this study, we exploited the utilization of DES of ZnCl_2_ and urea in accelerating the reaction of thiazolidinone analogues with variedly substituted benzaldehydes to prepare the corresponding condensed products. The DES acted as a solvent as well as a catalyst in these reactions. This is the first report in which the condensation of aldehydes with rhodanine, as well as thiazolidine‐2,4‐dione using DES made from ZnCl_2_ and urea, has been carried out, to the best of our knowledge. The present strategy offers remarkable advantages such as quick reaction time, excellent reaction yields, mild reaction conditions, high functional group tolerance, column‐free isolation, recyclability, and usability of catalyst.

## Results and Discussion

### Synthesis

Our preliminary studies employed a model reaction between benzaldehyde **1 a** (1 eq.) and rhodanine **2 a** (1 eq.) in ZnCl_2_/urea DES as depicted in Scheme 1. Table 1 describes different reaction conditions (solvent, temperature, yield, etc) explored in optimizing the model reaction.

**Table 1:** The optimization of protocols for the synthesis of target product.

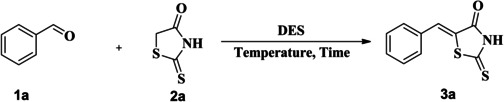					
**Scheme 1**					
Entry	DES	Solvent	Temperature (°C)	Time (min)	Yield^[a]^ (%)
1	–	–	80	60	20
2	ZnCl_2_/urea (1 : 3.5)	–	80	10	90
3	ZnCl_2_/urea (1 : 3.5)	–	100	2	95
**4**	**ZnCl_2_/urea (1 : 3.5)**	–	**120**	**2**	**98**
5	ZnCl_2_/urea (1 : 3.5)	–	140	2	85
6	ChCl/malonic acid (1 : 2)	–	120	60	34
7	ChCl/glycerol (1 : 2)	–	120	60	70
8	ChCl/urea (1 : 2)	–	120	60	75
9	ChCl/ZnCl_2_ (1 : 2)	–	120	60	68
10	ChCl/SnCl_2_ (1 : 2)	–	120	60	46
11	ZnCl_2_	–	120	30	80
12	Urea	–	120	30	90
13	FeCl_3_	–	120	30	60
14	AlCl_3_	–	120	30	68
15	ZnCl_2_/urea (1 : 3.5)	EtOH	reflux	60	70
16	ZnCl_2_/urea (1 : 3.5)	CH_3_CN	reflux	60	55
17	ZnCl_2_/urea (1 : 3.5)	DCM	reflux	60	40

[a] Isolated Yield.

First, heating the reactants at 80 °C for 60 minutes afforded **3 a** in 20 % yield (Entry 1, Table 1). The performance of different DESs *viz*. [ZnCl_2_/urea (1 : 3.5), ChCl/malonic acid (1 : 2), ChCl/glycerol (1 : 2), ChCl/urea (1 : 2), ChCl/ZnCl_2_ (1 : 2) and ChCl/SnCl_2_ (1 : 2)] at different temperatures was subsequently investigated (Entry 2–10, Table 1). Notably, the ZnCl_2_/urea DES offered the best yield (98 %) of the desired product within 2 minutes (Entry 4, Table 1). However, the catalytic efficiency of this DES decreased when the temperature was below or above 120 °C, thus compromising the product yield (Entry 2,3 and 5, Table 1).

To assess the individual role of different DES components, we performed the model reaction in the presence of ZnCl_2_, urea, SnCl_2_, FeCl_3,_ or AlCl_3_ (Entry 11–14, Table 1) at 120 °C. Although all these Lewis acids managed to promote the reaction to a reasonable extent, the reaction time was several folds higher than that of ZnCl_2_/urea DES including compromisation of the yield in most cases. These results suggest that the stronger hydrogen bond forming ability of ZnCl_2_/urea DES is favorable for more efficient reaction conversion. Furthermore, the efficiency of ZnCl_2_/Urea DES for the same reaction in different solvents (ethanol, acetonitrile, and DCM) was also examined (Entry 15–17, Table 1). Again, these solvents negatively impacted the product yields (40–70 %), including much longer reaction time. Overall, for the model reaction, the heating at 120 °C in ZnCl_2_/Urea (1 : 3.5) DES was found to be the best reaction condition and was subsequently employed for further derivatization of **3 a** by using a variety of aromatic aldehydes.

Table 2 shows the chemical structures of different thioxothiazolidin‐4‐ones (**3 a**–**u**) synthesized from the condensation reaction between rhodanine and different benzaldehydes bearing both electron‐donating (ED) and electron‐withdrawing (EW) groups. Generally, both ED and EW groups were well tolerated in these chemical transformations yielding the majority of the corresponding products within 2 minutes with good to excellent yields (88–99 %). Notably, the aromatic aldehydes bearing ED groups (CH_3_, and OCH_3_) at *meta* position (**3 h**, and **3 i**) reacted more quickly. They provided higher yields than the ED groups at *ortho* (**3 m**, and **3 n**) and *para* (**3 b**, and **3 c**) positions attributed probably due to the resonance effect of the substituents probably attributed to the resonance effect of the former substituents increase the electrophilicity of the carbonyl group towards the nucleophilic attack. It was further corroborated by the fact that the aldehydes bearing EW groups (F, Cl, and Br) at *ortho* (**3 o**, **3 p**, and **3 q**) and *para* (**3 e**, **3 f**, and **3 g**) positions were more reactive and provided higher yields when compared with their *meta* (**3 j**, **3 k**, and **3 l**) counterparts. Also, the aromatic aldehydes bearing more than one EW group offered higher yields as compared to the ED groups (**3 r**, **3 t**, and **3 u)**. Finally, the tri‐substituted aldehydes offered a slightly lower yield (**3 r**) as compared to their disubstituted counterparts (**3 t** and **3 u**) probably due to steric factors.

**Table 2:**
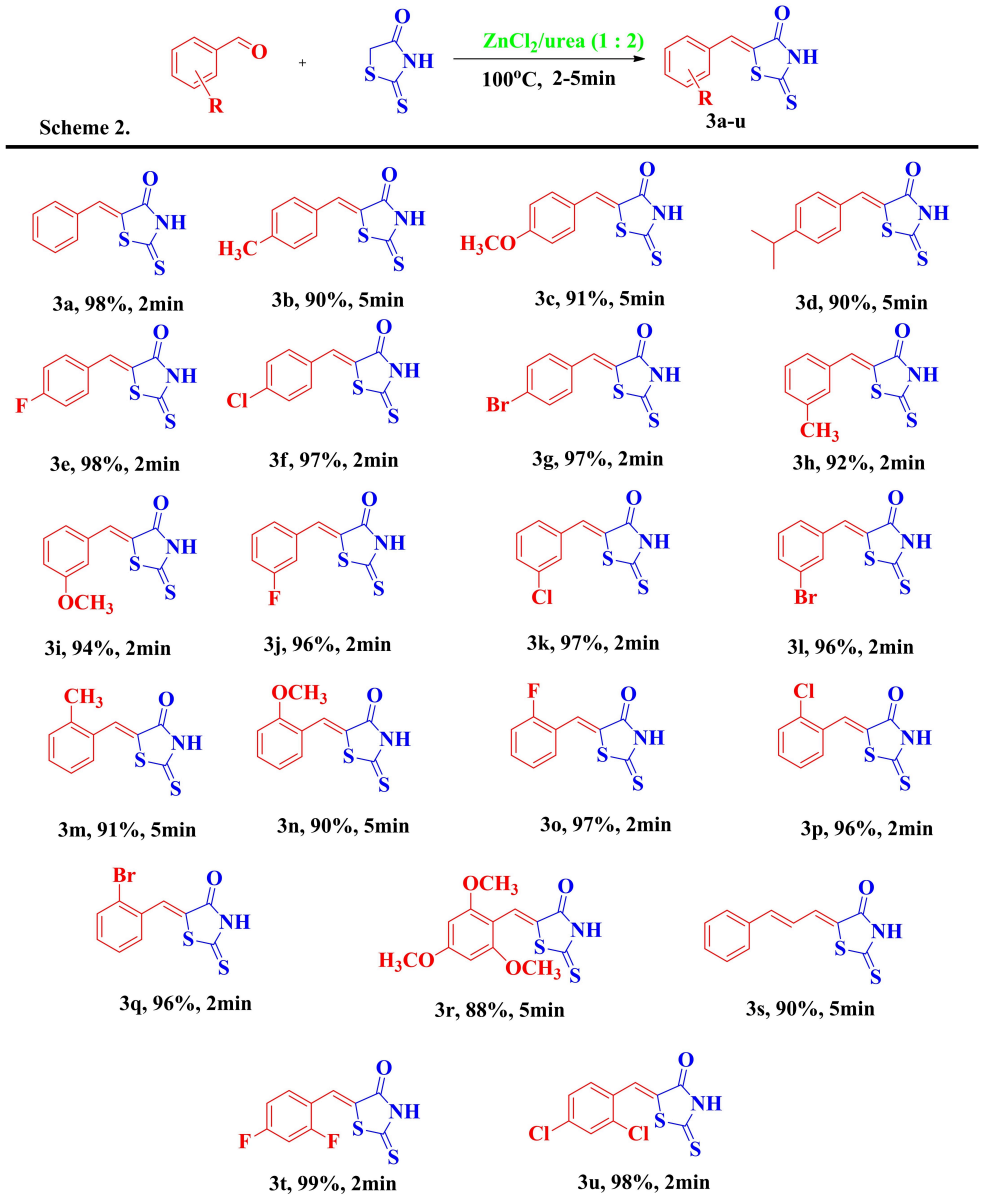
The scope of different aldehydes with rhodanine.

To extend the scope of this methodology, we also reacted thiazolidine‐2‐one with different aldehydes to prepare the corresponding adducts, as depicted in Table 3. Again, the majority of the final compounds formed in 2 minutes demonstrate the high reaction conversion efficiency of the methodology developed. Again, the electron‐donating groups at the para position of benzaldehydes (**4 b** and **4 c**) lowered the reaction yields compared with those bearing electron‐withdrawing substituents at the same position (**4 d** and **4 e**).

**Table 3:**
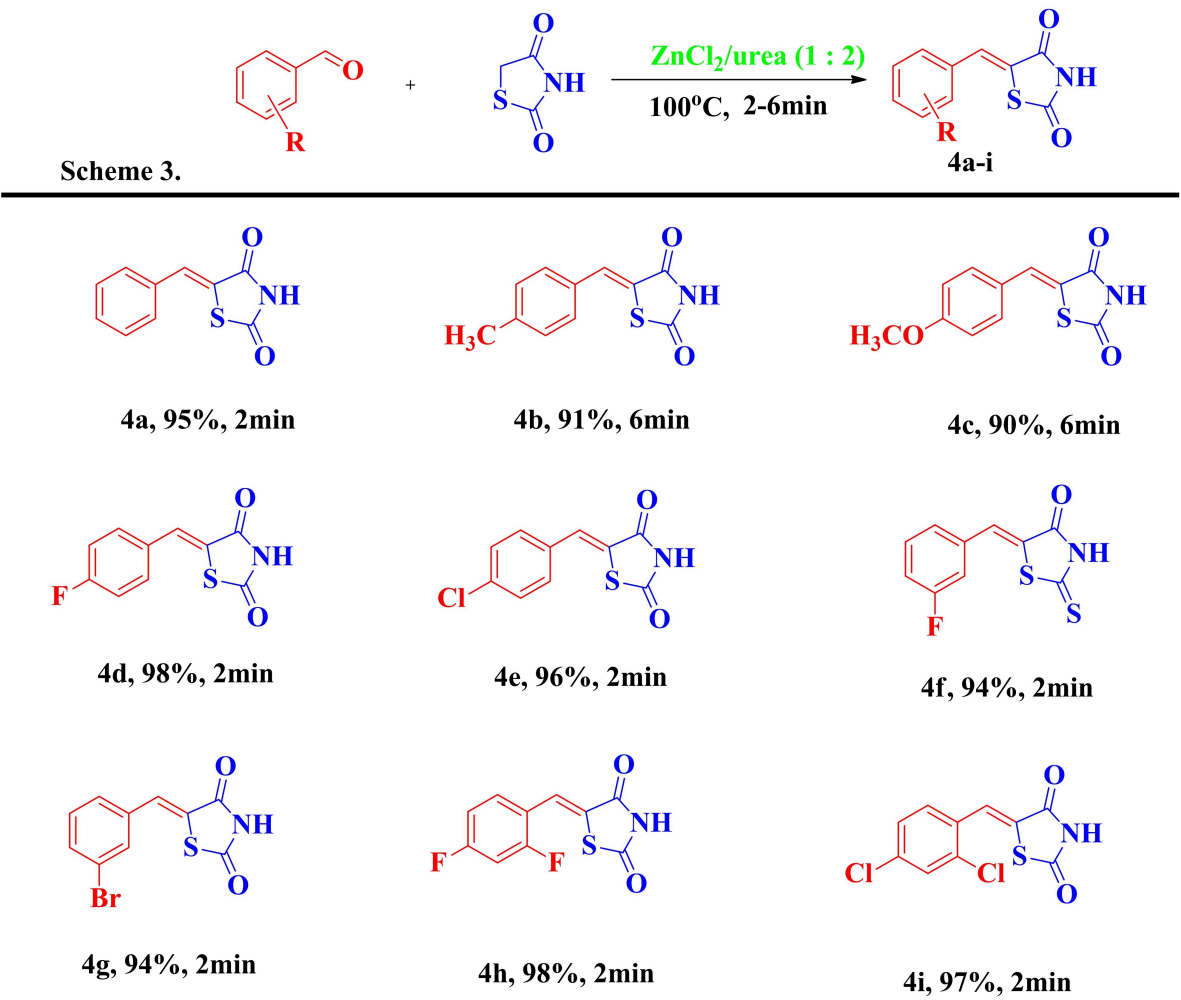
The scope of different aldehydes with thiazolidine‐2,4‐dione.

The comparison of Tables 2 and 3 also revealed that the condensation reactions of rhodanine and thiazolidine‐2,4‐dione with aldehydes generally took place within 2 minutes with a few exceptions.

**Figure 2:**
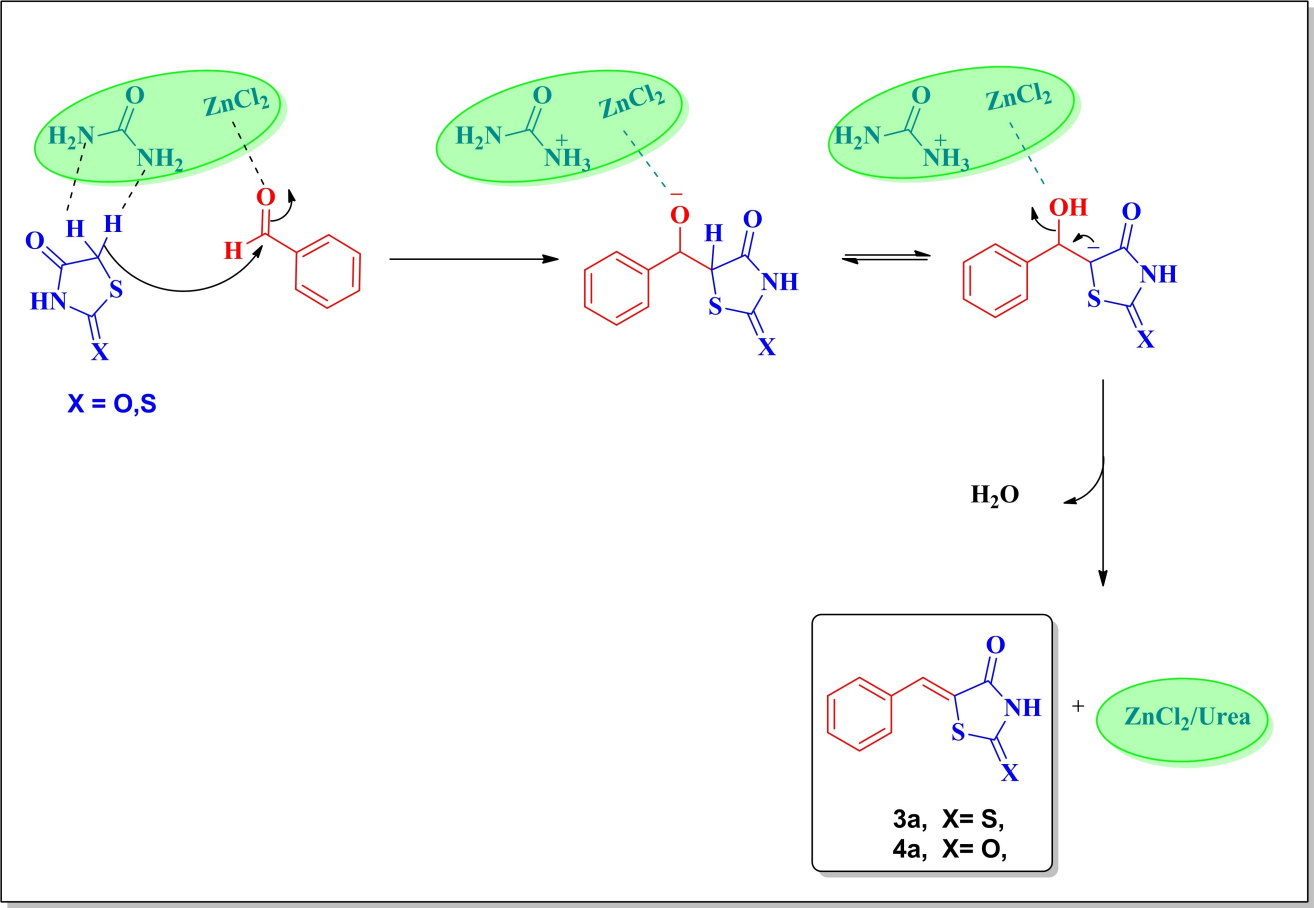
A plausible mechanism for the condensation of aldehyde with rhodanine and thiazolidin‐2‐one in the presence of DES.

In accordance with previous studies, a plausible mechanism depicting the activation of the starting materials by ZnCl_2_/Urea DES has been proposed and pictorially shown in Figure 2.

As it can be seen ZnCl_2_/Urea DES plays not only a role as a solvent but also exhibits catalytic properties attributable to its robust hydrogen bond network and ionic environment. These attributes effectively reduce the activation energy barrier of the reaction process by solvating the ionic transition state. Initially, activation of the aldehyde and rhodanine occurs via hydrogen bonding interactions facilitated by DES, promoting their condensation reaction and yielding an enol intermediate. Subsequent Knoevenagel condensation between this enol intermediate and activated aldehyde results in the formation of the thiazolidinones, accompanied by water elimination.

### Gram Scale Synthesis

To demonstrate the applicability of the reaction methodology at a large scale. The condensation reactions between benzaldehyde and rhodanine or thiazolidin‐2‐one were conducted. Specifically, the preparation of (Z)‐5‐benzylidene‐2‐thioxothiazolidin‐4‐one product **3 a** at gram scale (98 % yield) was achieved by reacting benzaldehyde (1.06 g, 10 mmol) and rhodanine (1.33 g, 10 mmol) in ZnCl_2_/urea (1 : 3.5) DES under heating at 120 °C as shown in Scheme 4. Similarly, the gram‐scale synthesis of (Z)‐5‐benzylidenethiazolidine‐2,4‐dione product **4 b** using benzaldehyde (1.06 g, 10 mmol) and thiazolidin‐2‐one (1.17 g, 10 mmol) in ZnCl_2_/urea (1 : 3.5) under heating at 120 °C, was also successfully carried out (Scheme 4). These results suggest the methodology's suitability and efficiency in producing the target compounds’ high‐scale production.

### Green Metrics Analysis

The green metrics analysis is utilized to assess the environmental friendliness of synthetic protocols based on different green metrics parameters such as atom efficiency (AEf), optimal efficiency (OE), atom economy (AE), reaction mass efficiency (RME), carbon efficiency (CE), water intensity (SI), mass productivity (MP), process mass intensity (PMI), E factor, and solvent intensity (SI) parameters. To determine the environmentally friendly nature of our reaction protocol, we performed the green metrics analysis of our reaction methodology for synthesizing compounds **3 a** and **4 b** and compared it with the two reported methods.[^45^, ^46^] The results obtained from this analysis are depicted in Tables 4 and 5[^28^, ^30^] while the details of the corresponding calculations are presented in the supporting information.

An examination of the results showed that our methodology (method A) outperforms the previously reported methods for the same compound (method B^[53]^ and method C^[54]^) as all the green metrics values for our method were better when compared to methods B and C (Tables 4 and 5). Notably, the values for AE, AEf, CE, RME, OE, and MP in our method were closer to 100 %, indicating a more environmentally friendly protocol (Table 4). Also, the lower values of PMI, E‐factor, SI, and WI further confirmed the greener nature of our method (Table 5).

**Table 4:** Green metrics (AE, CE, AEf, RME, MP, and OE) for **3 a**, and **4 b**.^[a]^

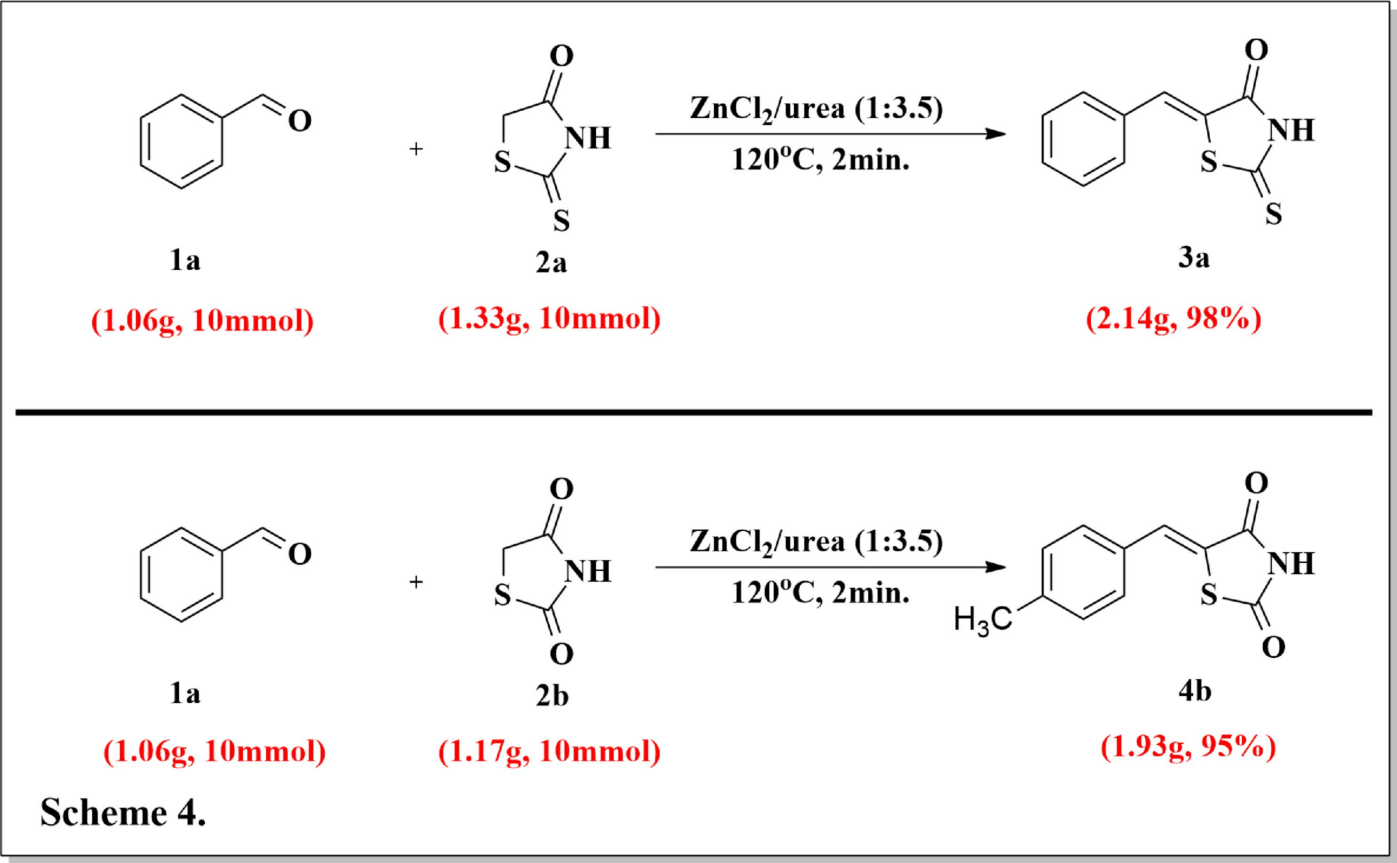								
Process	No. of steps	Yield (%)	AE (%)	AEf (%)	CE (%)	RME (%)	OE (%)	MP (%)
**Compound 3 a**								
Method A (this work)	1	98	92.47	90.62	98	90.5	97.86	3.16
Method B (ref. [45])	1	95	92.47	87.84	95	87.73	94.87	2.67
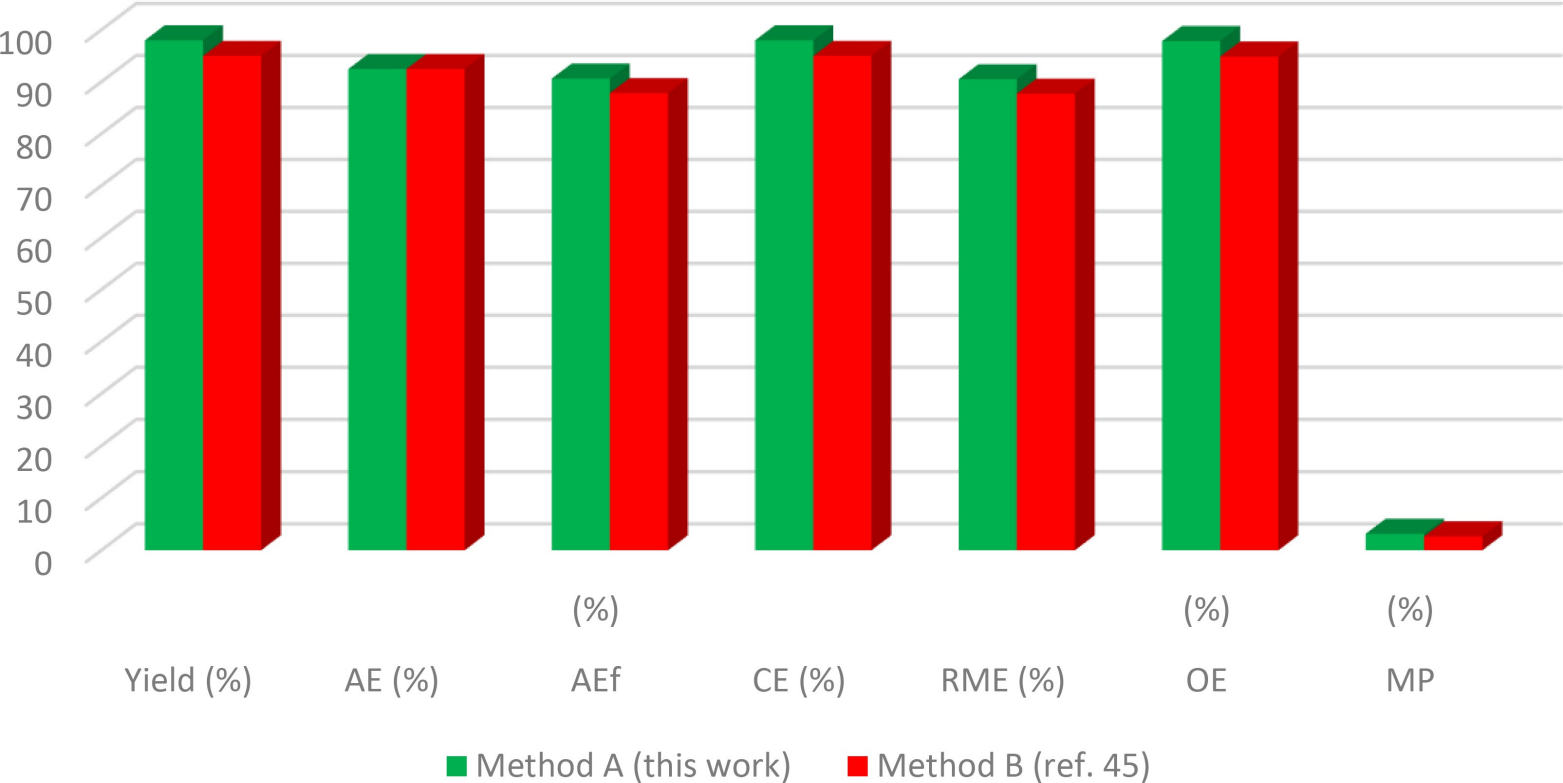								
**Compound 4 b**								
Method A (this work)	1	91	92.33	84.02	91	84.01	90.98	2.91
Method C (ref. [46])	1	75	92.33	69.24	75	69.24	74.99	1.53
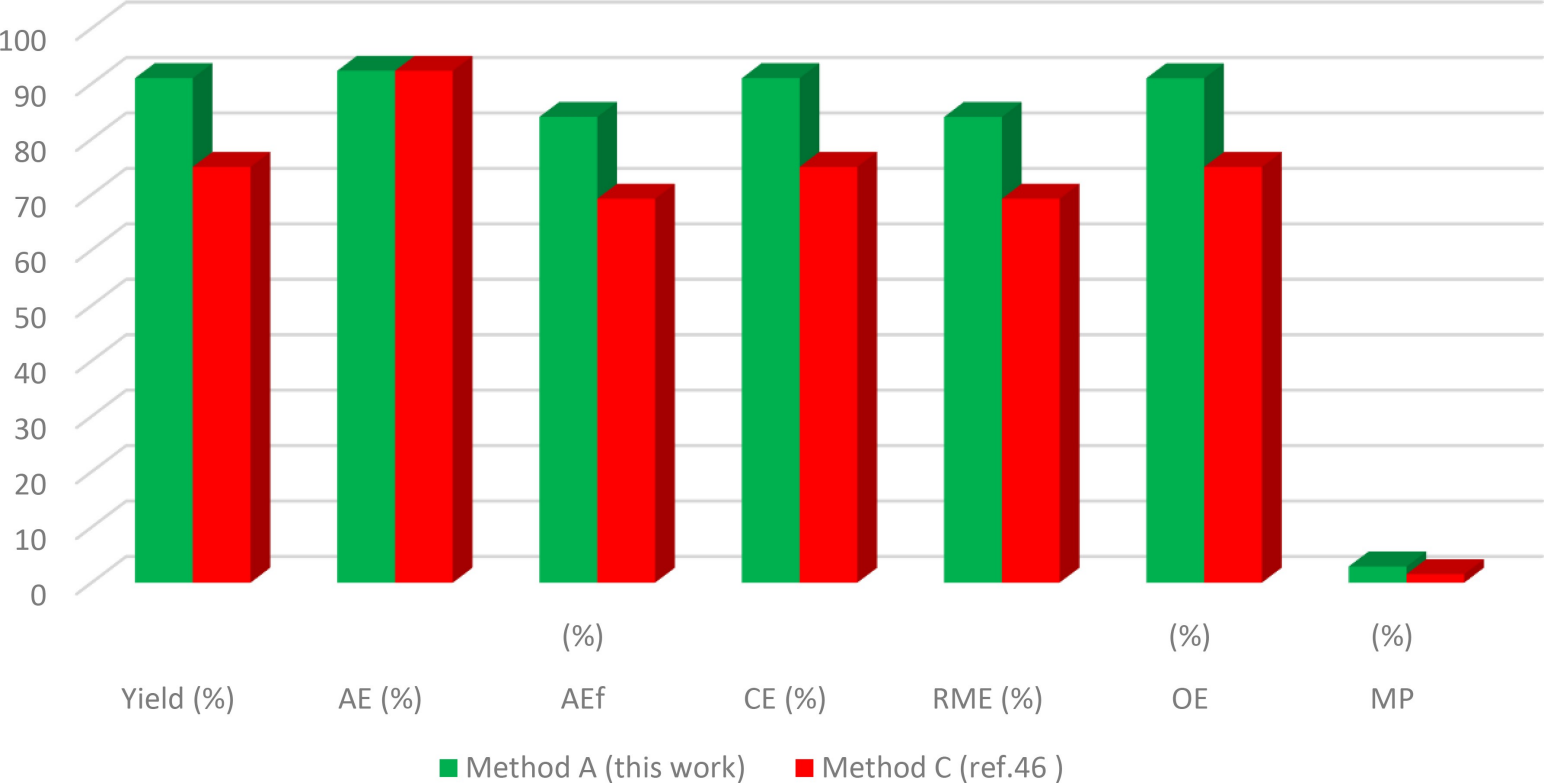								

[a] Value closer to 100 % have a more greener and more eco‐friendly process. More value of AE, AEf, CE, RME, OE, and MP indicated that methodology follows the green protocols and plays a significant role in green chemistry.

**Table 5:** Green metrics (PMI, SI, E factor, and WI) for **3 a**, and **4 b**.^[a]^

Process	PMI (g/g)	E factor (g/g)	SI (g/g)	WI (g/g)
Compound **3 a**				
Method A (this work)	31.58	30.58	7.39	23.08
Method B (ref. [45])	37.42	36.42	24.42	7.14
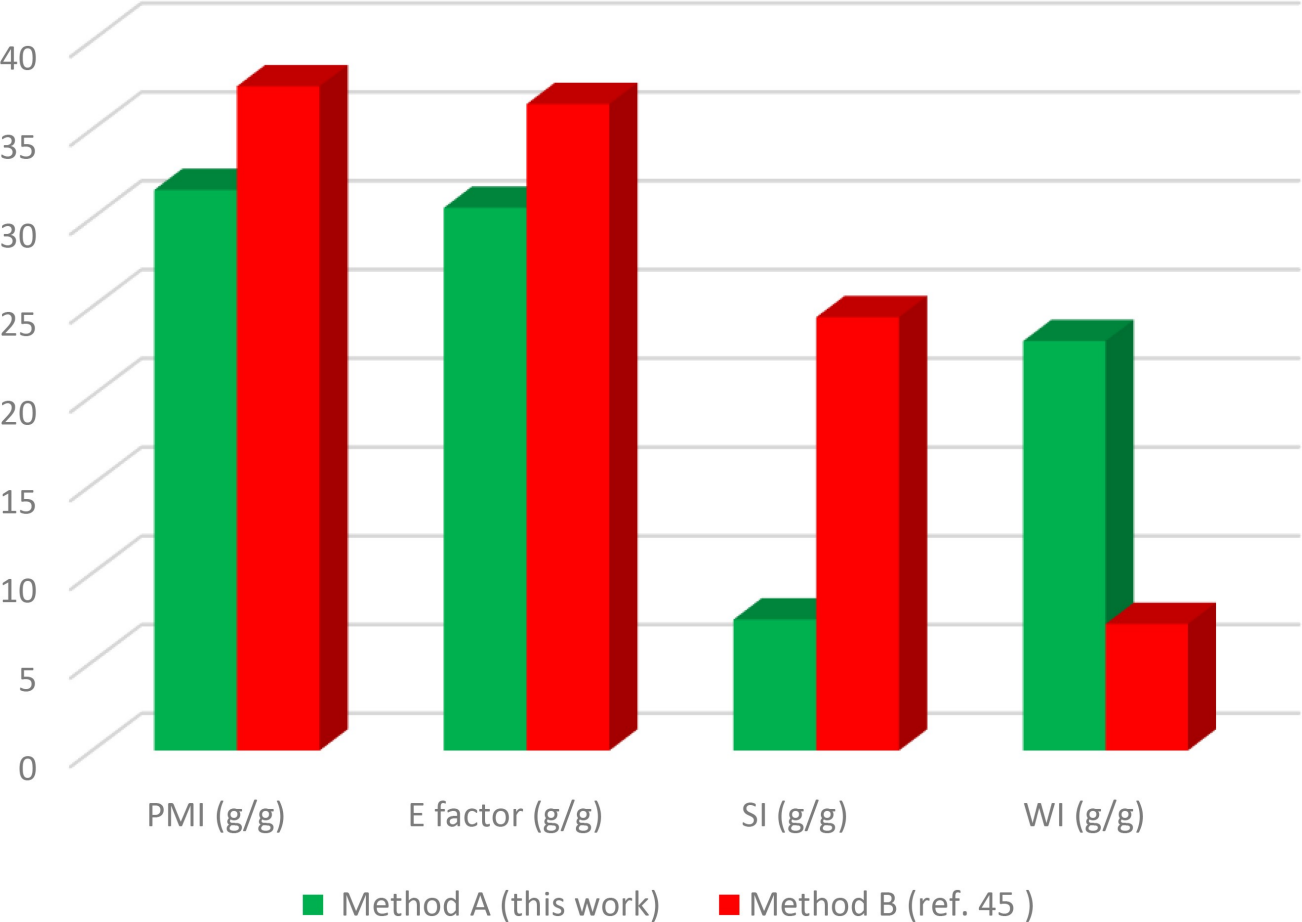				
Compound **4 b**				
Method A (this work)	34.31	33.31	8.03	25.08
Method C (ref. [46])	65.27	64.27	34.39	30.44
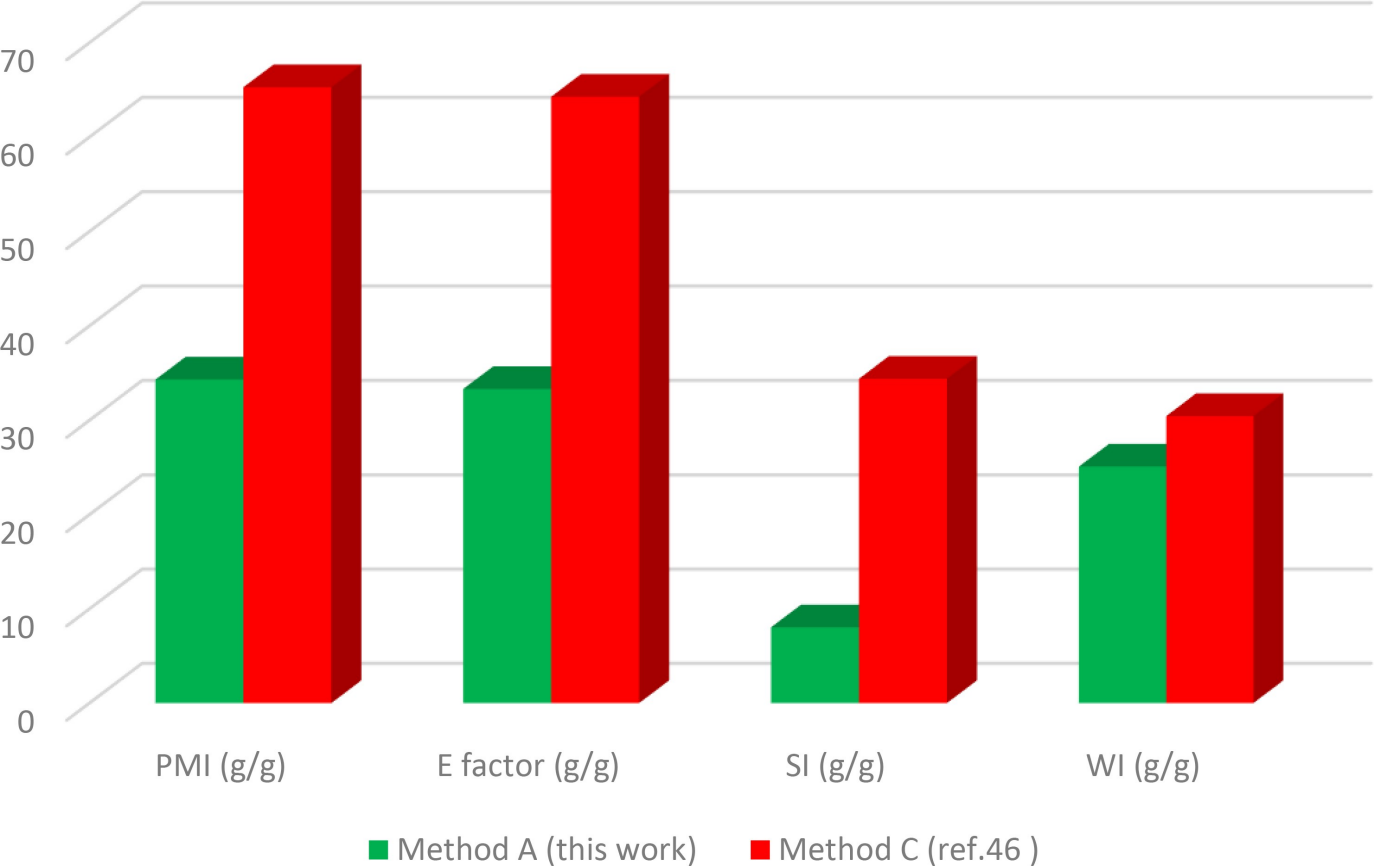				

[a] better reaction protocols have a lower value.

### Recyclability and Reusability of DES

**Figure 3:**
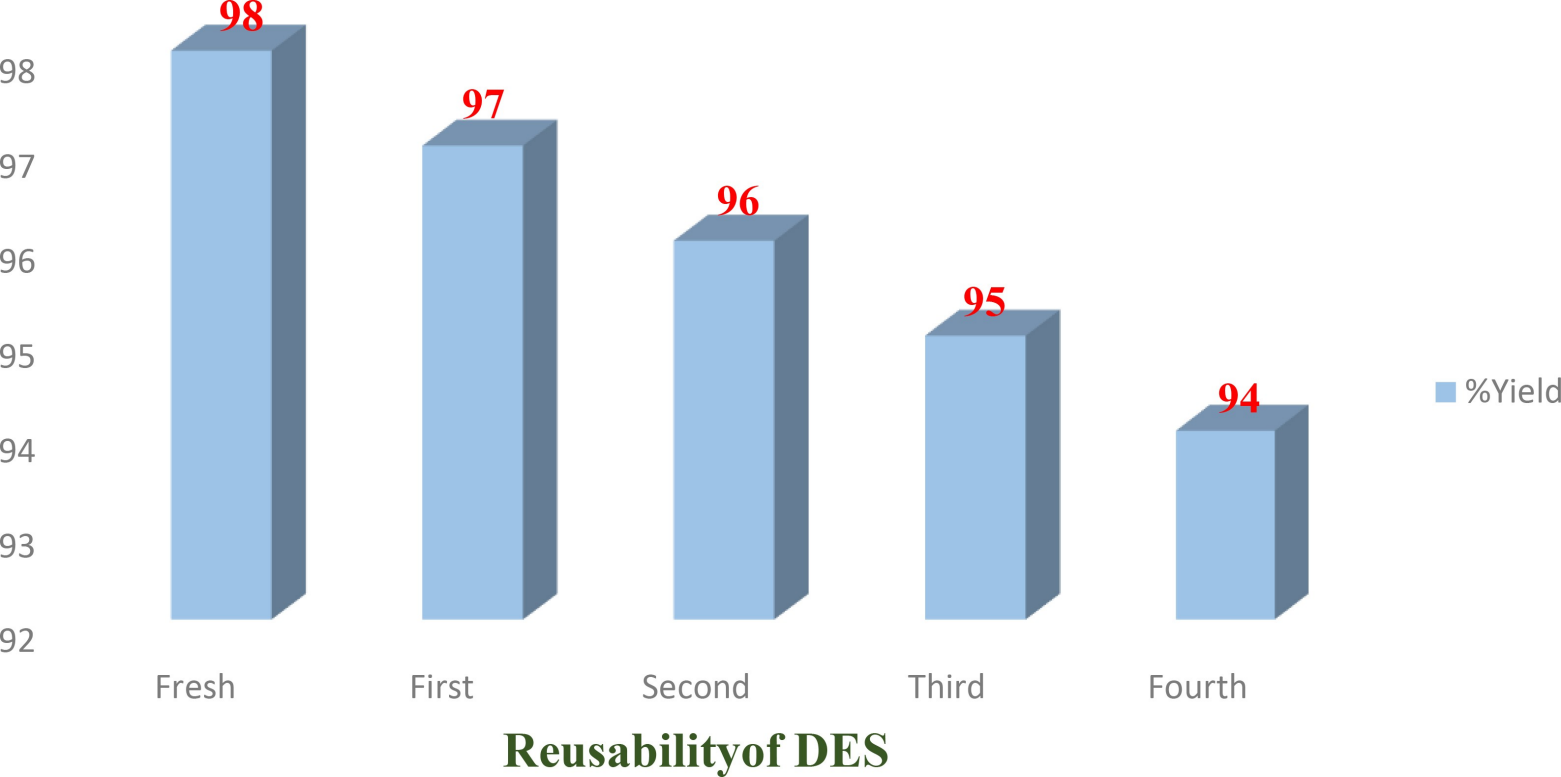
Recyclability of ZnCl_2_/urea DES.

DESs in addition to being greener and environmentally friendly, should be reusable and recyclable to develop an economic and cost‐effective methodology. We determined the efficiency of ZnCl_2_/urea DES by executing a chemical reaction between benzaldehyde and rhodanine, and subsequently recovering it by adding water to the reaction mixture and filtering the solid product before reusing it for the same reaction. The results are shown in Figure 3. It can be observed that the DES was able to produce the target compound in good yields (>90 %) even after using it four times to carry out the same reaction (Figure 3), and thus demonstrates the high reusability of the DES investigated in this study.

Table 6 compares the reaction conditions/outcome of the previously published work directed in the synthesis of (Z)‐5‐benzylidene‐2‐thioxothiazolidin‐4‐one analogues with the present work and demonstrates the significance of the present study.

**Table 6:** Comparison studies with previously reported methods.

Entry	Author^R^	Catalyst	Solvent/Energy	Reaction Time	Yields
1	Khazaei^[53]^	Bu_4_NOH	H_2_O‐EtOH/50 °C	1.5 h	85–95 %
2	Gadekar^[51]^	TS‐1	H_2_O/90 °C	30 min	85–92 %
3	Mahalle^[54]^	PEG‐300	130 °C	3 h	75–84 %
4	Shelke^[48]^	KAl(SO_4_)_2_.12H_2_O	H_2_O/90°C	1–1.5 h	85–95 %
5	Schan^[55]^	Piperidine	EtOH/80 °C	4 h	51–90 %
6	Veisi^[52]^	SBA‐15‐PrNH_2_	EtOH/50 °C	2 h	48–95 %
7	Salem^[56]^	NaOAC	CH_3_COOH/120 °C	3–4 h	90 %
8	Jinju^[49]^	Tween80, K_2_CO_3_	H_2_0/rt	3–66 h	66–95 %
9	Wang^[50]^	I_2_	Grinding	5–10 min	88–95 %
10	Paiva^[57]^	Urea	100 °C	2 h	45–99 %
**11**	**Present**	**DES**	–	**2–6 min**	**88–99 %**

^R^Reference

## Density Functional Theory (DFT) Studies

We further explored different reactivity and stability parameters of all our compounds at DFT level and the results are summarized in the following sections.

### Frontier Molecular Orbital (FMO) Analysis

Frontier Molecular Orbitals (FMOs) play a fundamental role in understanding the chemical stability, electronic properties, and optical properties of molecules.[^58^, ^59^, ^60^]

**Figure 4:**
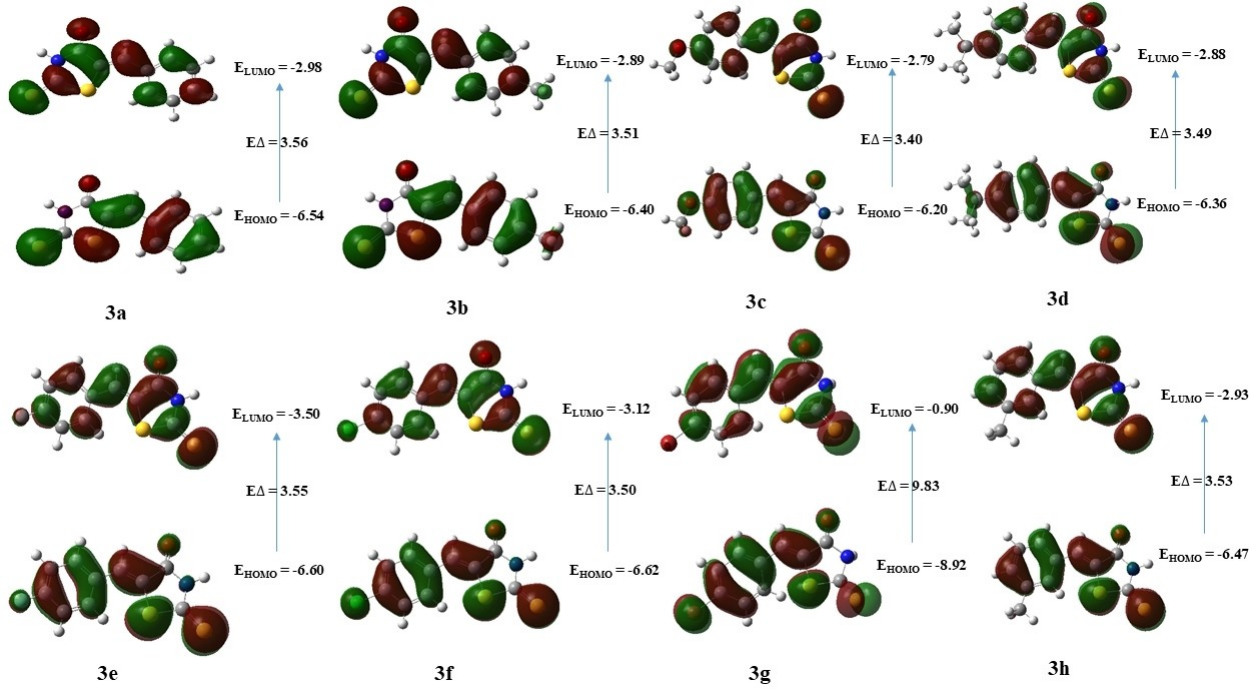
Illustration of the FMOs of compounds **3 a**–**3 h**.

**Figure 5:**
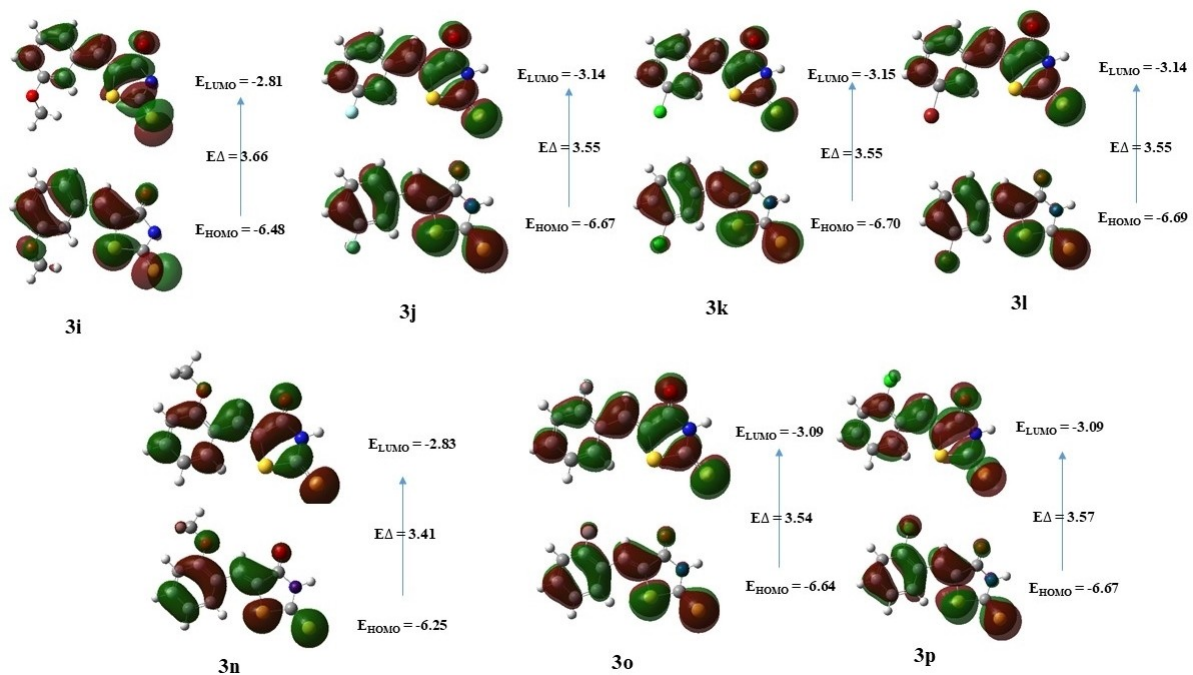
Illustration of FMOs of compounds **3 i**–**3 l** & **3 n**–**3 p**.

**Figure 6:**
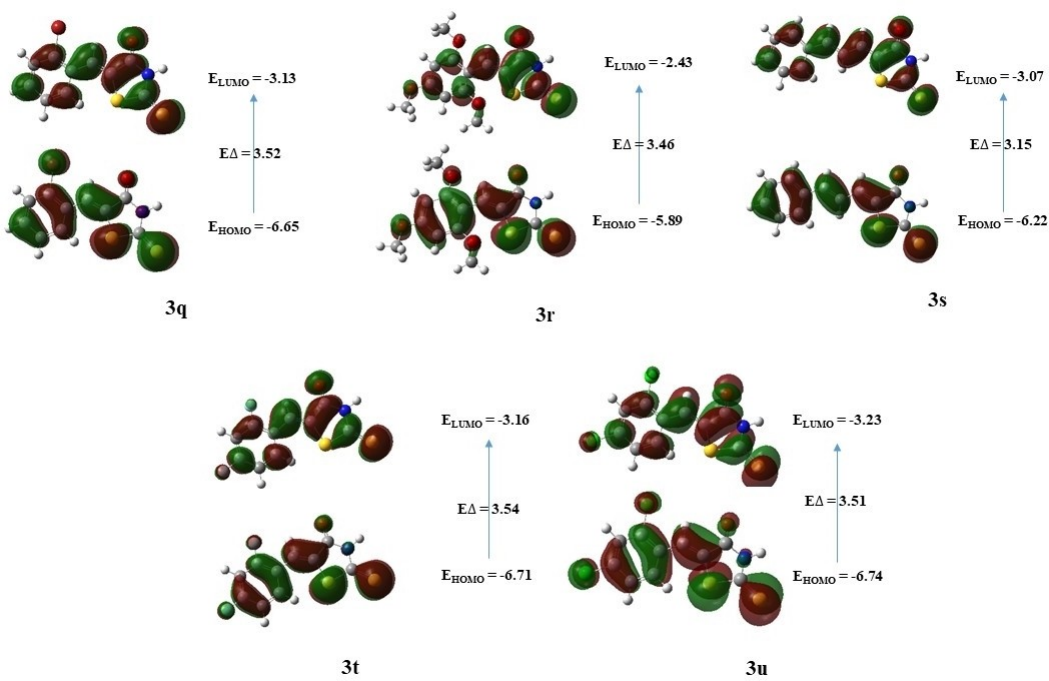
Illustration of FMOs of compounds **3 q**–**3 u**.

FMOs are used to interpret molecular structure and reactivity of organic and metal complexes.^[61]^ These molecular orbitals consist of the Highest Occupied Molecular Orbital (HOMO), representing the highest‐energy occupied orbital, and the Lowest Unoccupied Molecular Orbital (LUMO), denoting the lowest‐energy unoccupied orbital.^[62]^ We computed the energies of the compounds (**3a‐u**) at the DFT level using a 6‐311++G (d, p) basis set and the results are summarized in Table 7. As can be seen that the compound **3 r** (E_HOMO_=−5.89 eV) has the highest HOMO among all other compounds. While compound **3 g** (E_HOMO_=−8.92 eV) has the lowest HOMO as compared to other compounds. On comparing the position of the methoxy group at *ortho* (**3 n**), *meta* (**3 i**), and *para (* **3 c**), the decreasing order HOMO energy was *para*>*ortho*>*meta* (E_HOMO_=−6.20>−6.25>−6.48 eV respectively). Compound **3 g** (E_HOMO_=−0.90 eV) was found to have the highest LUMO energy, and compound **3 u** (E_HOMO_=−3.23 eV) with lowest LUMO energy. The energy gap, which reflects the difference between the HOMO and LUMO energies, has significant implications for chemical reactivity.[^63^, ^64^] A smaller energy gap suggests increased reactivity, while a larger energy gap indicates decreased reactivity or increased stability.^[65]^ For compound **3 g**, the computed energy gap between HOMO and LUMO was large (ΔE =9.83 eV) as compared to the other compounds. On the other hand, compound **3 s** was found to have the smallest energy gap (ΔE =3.15 eV), suggesting high stability profile of the former. Figures 4, 5, and 6 represent the distribution of charge density on the HOMO and LUMO as well as the energy gaps of all compounds studied at DFT level.

**Table 7:** The FMOs energies of the synthesized compounds.

Properties	E_HOMO_ (eV)	E_LUMO_ (eV)	Δ E (eV)
Code			
**3 a**	−6.54	−2.98	3.56
**3 b**	−6.40	−2.89	3.51
**3 c**	−6.20	−2.79	3.40
**3 d**	−6.38	−2.88	3.49
**3 e**	−6.60	−3.05	3.55
**3 f**	−6.62	−3.12	3.50
**3 g**	−8.92	−0.90	9.83
**3 h**	−6.47	−2.93	3.53
**3 i**	−6.48	−2.81	3.66
**3 j**	−6.67	−3.14	3.55
**3 k**	−6.70	−3.15	3.55
**3 l**	−6.69	−3.14	3.54
**3 n**	−6.25	−2.83	3.41
**3 o**	−6.64	−3.09	3.54
**3 p**	−6.67	−3.09	3.57
**3 q**	−6.65	−3.13	3.52
**3 r**	−5.89	−2.43	3.46
**3 s**	−6.22	−3.07	3.15
**3 t**	−6.71	−3.16	3.54
**3 u**	−6.74	−3.23	3.51

Table 8 shows the global reactivity parameters such as ionization potential (**I**), electron affinity (**A**), hardness (**η**), electronegativity (**χ**), softness (**S**) chemical potential (**μ**), and electrophilicity index (**ω**) of the synthesized compounds (**3a‐u**) predicted at the DFT level.[^66^, ^67^, ^68^]

**Table 8:** The global reactivity properties of the synthesized compounds.

Properties	I (eV)	A (eV)	η (eV)	χ (eV)	S (eV^−1^)	μ (eV)	ω (eV)
Code							
**3 a**	6.54	2.98	1.78	3.32	0.15	−3.32	3.10
**3 b**	6.40	2.89	1.76	3.25	0.15	−3.25	3.01
**3 c**	6.20	2.79	1.70	3.15	0.16	−3.15	2.92
**3 d**	6.38	2.88	1.75	3.24	0.15	−3.24	3.01
**3 e**	6.60	3.05	1.77	3.35	0.15	−3.35	3.17
**3 f**	6.62	3.12	1.75	3.36	0.15	−3.36	3.24
**3 g**	8.92	0.90	4.91	4.44	0.11	−4.44	2.00
**3 h**	6.47	2.93	1.769	3.29	0.15	−3.29	3.06
**3 i**	6.48	2.81	1.83	3.29	0.15	−3.29	2.90
**3 j**	6.67	3.14	1.78	3.40	0.15	−3.40	3.26
**3 k**	6.70	3.15	1.78	3.40	0.15	−3.40	3.26
**3 l**	6.69	3.14	1.77	3.40	0.15	−3.40	3.26
**3 n**	6.25	2.83	1.70	3.17	0.16	−3.17	2.95
**3 o**	6.64	3.09	1.77	3.37	0.15	−3.37	3.221
**3 p**	6.67	3.09	1.78	3.39	0.15	−3.39	3.22
**3 q**	6.65	3.13	1.76	3.38	0.15	−3.38	3.25
**3 r**	5.89	2.43	1.73	2.99	0.17	−2.99	2.58
**3 s**	6.22	3.07	1.57	3.16	0.16	−3.16	3.18
**3 t**	6.71	3.16	1.77	3.41	0.15	−3.41	3.28
**3 u**	6.74	3.23	1.75	3.43	0.15	−3.43	3.35

Ionization potential (**I**) and electron affinity (**A**) values provide insights into the molecule's tendency to lose or gain electrons, respectively.^[69]^ The I value is related to E_HOMO_ (**I**=‐ E_HOMO_) and the A value is related to E_LUMO_ (**A**=−E_HOMO_), hence as the **I** value decreases, the electron removal becomes easier, while as the **A** value increases, electron addition becomes faster.^[70]^ Accordingly, compound **3 r** with lowest **I** value (**I**=5.89 eV), was predicted to be the strongest nucleophile while compound **3 s** with the highest **A** value (**A**=3.23 eV) was predicted to be the strongest electrophile.

Electronegativity (**χ**) is a parameter that describes an atom's or group of atoms’ ability to attract electrons toward itself.^[71]^ The compound **3 g** (4.44 eV) was predicted to have the highest electronegativity among all the compounds, while compound **3 r** (2.99) had the lowest electronegativity. Chemical potential (**μ**) is linked to electronegativity **(χ**) and measures the electrons’ propensity to be attracted toward each other within a molecule.^[72]^

Global Hardness (**η**) quantifies the molecule's resistance to charge transfer, whereas global Softness (**S**) gauges its propensity for charge transfer.^[73]^ It is desirable for a molecule to possess lower global hardness and higher global softness, as this facilitates enhanced charge transfer to nearby biomolecules, thereby increasing interaction.^[58]^ The compound **3 s** (η=1.57 eV and S=0.16 eV^−1^) was computationally found to show better interaction with nearby molecules or biomolecules.

The Electrophilicity Index (ω) evaluates a molecule's ability to attract electrons, shedding light on the energy of stabilization when the molecule interacts with nearby electrons from its environment.^[74]^

**Figure 7:**
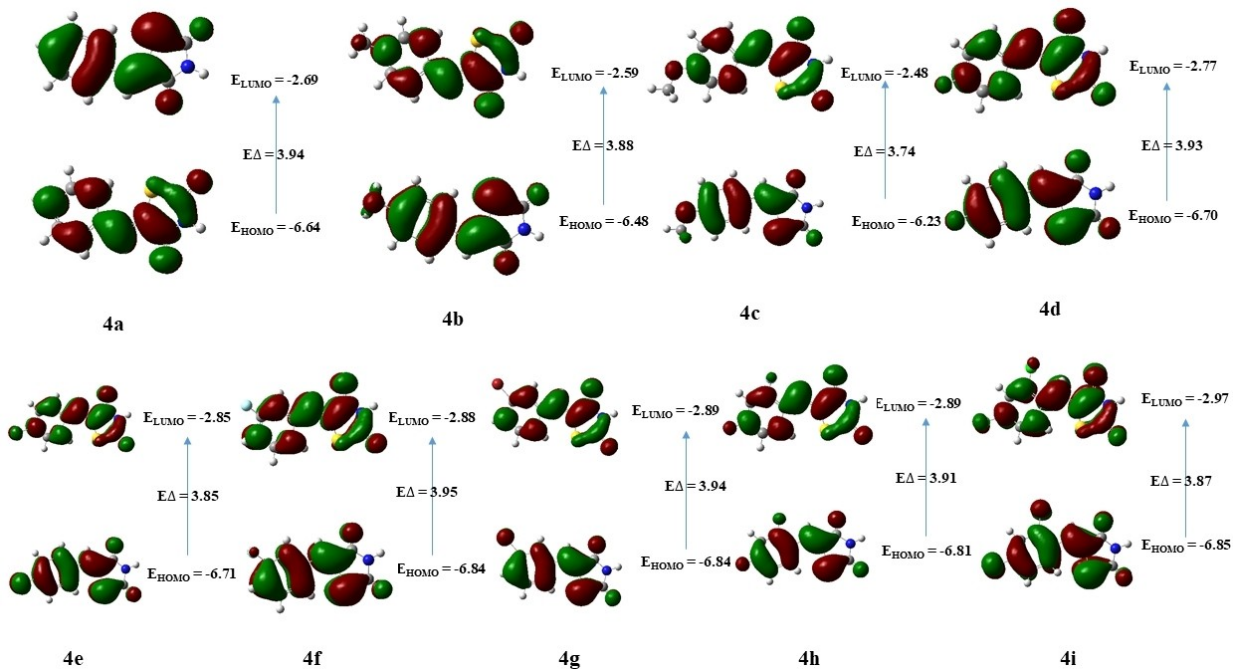
The FMOs orbitals HOMO and LUMO of compounds **4 a**–**4 i**.

The FMOs calculation were also performed for compounds **4 a**–**4 i**, and the results are depicted in Table 9. Computational results indicated that compound **4 c** has the highest HOMO (E_HOMO_=−6.23 eV) as well as the highest LUMO (E_HOMO_=−2.48 eV) energy among all other compounds while compound **4 i** has shown the lowest HOMO (E_HOMO_=−6.85 eV) and lowest LUMO (E_HOMO_=−2.97 eV) energies. The largest predicted energy gap between HOMO and LUMO suggested compound **4 a** (Δ E=3.94 eV) to be highly stable as compared to other derivatives. Compound **4 c** on the other hand was predicted to be the most reactive based on the smallest energy gap (Δ E=3.74 eV) in the series **4 a**–**4 i**. Figure 7, illustrates that the charge density of the HOMO and LUMO was distributed across the entire molecule in these compounds.

**Table 9:** The FMO energies of the synthesized compounds **4 a**–**4 i**.

Properties	E_HOMO_ (eV)	E_LUMO_ (eV)	Δ E (eV)
Code			
**4 a**	−6.64	−2.69	3.94
**4 b**	−6.48	−2.59	3.88
**4 c**	−6.23	−2.48	3.74
**4 d**	−6.70	−2.77	3.93
**4 e**	−6.71	−2.85	3.85
**4 f**	−6.84	−2.88	3.95
**4 g**	−6.84	−2.89	3.94
**4 h**	−6.81	−2.89	3.91
**4 i**	−6.85	−2.97	3.87

The global reactivity properties were also calculated for compounds **4 a**–**4 i**, and are depicted in Table 10. The calculated result shows that compound **4 c** has the lowest **I** value (2.48 eV) signifying the most accessible electron removal tendency (oxidation potential) of this compound, while compound **4 i** has the highest **A** value (2.97 eV), suggesting its excellent stability upon electron addition (reduction potential).

**Table 10:** The global reactivity properties of the synthesized compounds **4 a**–**4 i**.

Properties	I (eV)	A (eV)	η (eV)	χ (eV)	S (eV^−1^)	μ (eV)	ω (eV)
Code							
**4 a**	6.64	2.69	1.97	3.37	0.15	−3.37	2.87
**4 b**	6.48	2.59	1.94	3.28	0.15	−3.28	2.78
**4 c**	6.23	2.48	1.87	3.16	0.16	−3.16	2.67
**4 d**	6.70	2.77	1.96	3.40	0.15	−3.40	2.94
**4 e**	6.71	2.85	1.92	3.40	0.15	−3.40	3.01
**4 f**	6.84	2.88	1.97	3.47	0.14	−3.47	3.04
**4 g**	6.84	2.89	1.97	3.47	0.14	−3.47	3.05
**4 h**	6.81	2.89	1.95	3.46	0.14	−3.46	3.05
**4 i**	6.85	2.97	1.93	3.48	0.14	−3.48	3.12

On comparing the electronegativity of this series, compound **4 i** (3.48 eV) has been predicted to have the highest electronegativity among all the derivatives. The compound **4 c** (η=1.87 eV and S=0.16 eV^−1^) was predicted to show better interaction with nearby molecular or biomolecules. The computed global electrophilicity index (3.12 eV) further predicted **4 i** to have the greatest tendency to accept electrons among others.

## Experimental

### General Information

#### Chemicals and Reagents

All the experimental chemicals used in the present investigation were purchased from Sigma Aldrich Chemical Co. and CRD suppliers and used without any further purification. The majority of reactions were conducted under normal operating circumstances.

#### General Analytical Information

The reaction's progress was studied using TLC 60 F254 silica gel plates. The Bruker Avance‐III spectrometers (600 MHz and 150 MHz) were utilized for ^1^H and ^13^C NMR spectra with TMS serving as the internal standard. Chemical shifts are expressed in ppm, and coupling constants (J) are given in Hz. All substrates are established compounds with documented CAS numbers in the literature.

#### General Procedures for the Synthesis 3 a‐3 u and 4 a‐4 i

A mixture of benzaldehyde **1** (1.0 eq.) and rhodanine or thiazolidinone **2** (1.0 eq.) was stirred in the presence of DES ZnCl_2_/urea at 120 °C for 2–5 min. Upon the reaction completion, the resulting reaction mixture was cooled to room temperature and quenched by adding water. The precipitate was filtered out using a Buchner funnel *in vacuo*. The crude product was purified by recrystallization using ethanol.

#### Analytical Data

**3 a**. ^1^H‐NMR (600 MHz, DMSO‐d6): 7.47 (pseudo t, J=7.17 Hz, 1H), 7.52 (pseudo t, J=7.65 Hz, 2H), 7.59 (d, J=9.29 Hz, 3H); ^13^C‐NMR (150 MHz, DMSO‐d6): δ=127.15, 129.87, 130.83, 130.96, 131.24, 133.68, 197.23.

**4 a**. ^1^H‐NMR (600 MHz, DMSO‐d6): 7.32 (d, J=8.32 Hz, 1H), 7.40 (d, J=7.32 (d, J=8.32 Hz, 1H), 7.43 (t, J=7.14 Hz, 1H), 7.47 (t, J=7.75 Hz, 2H), 7.53 (d, J=7.44 Hz, 2H), 7.74 (s, 1H), 12.58 (s, 1H); ^13^C‐NMR 150 MHz, DMSO‐d6): δ=124.02, 129.70, 130.43, 130.79, 132.18, 133.47, 167.81, 168.35.

The analytical data of the other derivatives were provided in the supporting information.

#### Computational Details (DFT Studies)

The molecular structures of all the synthesized compounds were optimized using Gaussian 16 W software.^[75]^ The B3LYP/6‐311++G (d, p) basis set was employed to optimize the structure and further calculate the electronic properties of the synthesized compounds. The FMOs, HOMO and LUMO were visualized using Gauss View 6.0 software to understand the effect of substituents on the reactivity of compounds.

## Conclusions

An efficient methodology for condensing rhodanine or thiazolidine‐2,4‐dione nuclei has been developed in a ZnCl_2_/Urea DES. The advantages associated with this methodology include cost‐effectiveness, environmentally friendly conditions, high yields, rapid reaction times, column free and reusability of the catalyst. Moreover, these protocols were also applicable on a gram scale reaction. Additionally, DFT calculations were employed to determine the kinetic stability and reactivity of the synthesized compounds. Green metrics further revealed the greener nature of the present methodology. This study offered a highly versatile and valuable organic and pharmaceutical chemistry approach.

## Conflict of Interests

The authors declare no conflict of interest.

1

## Data Availability

The data that support the findings of this study are available in the supplementary material of this article.
